# Artificial Intelligence-based Segmentation of Residual Pancreatic Cancer in Resection Specimens Following Neoadjuvant Treatment (ISGPP-2): International Improvement and Validation Study

**DOI:** 10.1097/PAS.0000000000002270

**Published:** 2024-07-02

**Authors:** Boris V. Janssen, Bart Oteman, Mahsoem Ali, Pieter A. Valkema, Volkan Adsay, Olca Basturk, Deyali Chatterjee, Angela Chou, Stijn Crobach, Michael Doukas, Paul Drillenburg, Irene Esposito, Anthony J. Gill, Seung-Mo Hong, Casper Jansen, Mike Kliffen, Anubhav Mittal, Jas Samra, Marie-Louise F. van Velthuysen, Aslihan Yavas, Geert Kazemier, Joanne Verheij, Ewout Steyerberg, Marc G. Besselink, Huamin Wang, Caroline Verbeke, Arantza Fariña, Onno J. de Boer

**Affiliations:** Departments of * Surgery; † Pathology, Amsterdam UMC, location University of Amsterdam; ‡ Cancer Center Amsterdam; § Department of Surgery, Amsterdam UMC, location Vrije Universiteit; ‖‖ Department of Pathology, OLVG, Amsterdam; Departments of ‡‡ Pathology; ‖‖‖ Biomedical Data Sciences, Leiden University Medical Center, Leiden; §§ Department of Pathology, Erasmus Medical Center; ‡‡‡ Department of Pathology, Maasstad ziekenhuis, Rotterdam; *** Laboratorium Pathologie Oost-Nederland, Hengelo; ††† Department of Pathology, Medisch Spectrum Twente, Enschede, The Netherlands; ‖ Department of Pathology, Koc University and KUTTAM Research Center, Istanbul, Turkey; ¶ Department of Pathology, Memorial Sloan Kettering Cancer Center, New York, NY; # Department of Anatomical Pathology, University of Texas MD Anderson Cancer Center, Houston, TX; ** Cancer Diagnosis and Pathology Group, Kolling Institute of Medical Research, Royal North Shore Hospital, St Leonards, NSW, Australia; †† University of Sydney, Sydney, NSW, Australia; ¶¶ Institute of Pathology, Heinrich-Heine-University and University Hospital of Duesseldorf, Duesseldorf, Germany; ## Department of Pathology, Asan Medical Center, Seoul, Republic of Korea; §§§ Department of Surgery of Medical Research, Royal North Shore Hospital, St Leonards, NSW, Australia; ¶¶¶ Department of Pathology, Institute of Clinical Medicine, University of Oslo; ### Department of Pathology, Oslo University Hospital, Oslo, Norway

**Keywords:** pancreatic cancer, histopathology, tumor response scoring, neoadjuvant therapy, artificial intelligence, machine learning

## Abstract

Neoadjuvant therapy (NAT) has become routine in patients with borderline resectable pancreatic cancer. Pathologists examine pancreatic cancer resection specimens to evaluate the effect of NAT. However, an automated scoring system to objectively quantify residual pancreatic cancer (RPC) is currently lacking. Herein, we developed and validated the first automated segmentation model using artificial intelligence techniques to objectively quantify RPC. Digitized histopathological tissue slides were included from resected pancreatic cancer specimens from 14 centers in 7 countries in Europe, North America, Australia, and Asia. Four different scanner types were used: Philips (56%), Hamamatsu (27%), 3DHistech (10%), and Leica (7%). Regions of interest were annotated and classified as cancer, non-neoplastic pancreatic ducts, and others. A U-Net model was trained to detect RPC. Validation consisted of by-scanner internal-external cross-validation. Overall, 528 unique hematoxylin and eosin (H & E) slides from 528 patients were included. In the individual Philips, Hamamatsu, 3DHistech, and Leica scanner cross-validations, mean F1 scores of 0.81 (95% CI, 0.77-0.84), 0.80 (0.78-0.83), 0.76 (0.65-0.78), and 0.71 (0.65-0.78) were achieved, respectively. In the meta-analysis of the cross-validations, the mean F1 score was 0.78 (0.71-0.84). A final model was trained on the entire data set. This ISGPP model is the first segmentation model using artificial intelligence techniques to objectively quantify RPC following NAT. The internally-externally cross-validated model in this study demonstrated robust performance in detecting RPC in specimens. The ISGPP model, now made publically available, enables automated RPC segmentation and forms the basis for objective NAT response evaluation in pancreatic cancer.

Neoadjuvant therapy (NAT) has rapidly become a routine practice in patients with borderline resectable pancreatic ductal adenocarcinoma (PDAC).^1^ Several studies, including 2 randomized trials, have confirmed that NAT can improve survival and margin-negative resection rates.^2–4^ Histologic examination of pancreatic resection specimens after NAT can help evaluate the effects of the given treatment on the cancer cells, with 2 main purposes.^5,6^ First, this assessment can enable a comparison of the efficacy of different neoadjuvant regimes in clinical trials. Second, objective tumor response scoring (TRS) could guide the choice of adjuvant therapy in individual patients.

Over the past few decades, several histopathological TRS systems for PDAC have been proposed.^6^ However, during the 2019 Amsterdam international consensus meeting on histologic assessment of tumor response in resected pancreatic cancer after NAT, the International Study Group of Pancreatic Pathologists (ISGPP) stated that most TRS systems suffer from flawed reasoning, and/or lack objective definition criteria.^5^ This flawed reasoning regarding regression-based TRS systems refers to the fact that the tumor burden before therapy is unknown. Moreover, the tumor bed, which is considered a proxy for the original tumor burden, may be difficult to identify due to the presence of pancreatitis-like changes. These statements were illustrated in the recently published ISGPP-1 study,^7^ in which 18 expert gastrointestinal and pancreatic pathologists from 9 countries were unable to reliably identify therapy effects based on histomorphology only. In addition, the interobserver agreement for current TRS systems was found to be suboptimal based on the analysis of 23 expert pathologists. These findings support the recently published ISGPP proposal to score residual pancreatic cancer (RPC) rather than tumor regression following NAT and the call for new objective definitions. Artificial intelligence (AI) models have the potential to reduce the impact of subjectivity issues in current TRS systems by enabling objective quantification of RPC and providing tools for new objective definitions, thereby improving the evaluation of the response to NAT.

This study aimed to enhance and validate an AI-based segmentation model for RPC after NAT.^8^ To achieve this, data from multiple centers were incorporated, leading to improved generalizability of the model. The aim of the improved model, known as the ISGPP segmentation model, is to facilitate an objective assessment of tumor response by automating the identification and quantification of RPC following NAT.

## MATERIALS AND METHODS

### Data Collection

Archival hematoxylin and eosin (H & E)-stained tissue slides from resection specimens of patients with PDAC who received neoadjuvant treatment were selected for inclusion in the study. Patients who met the eligibility criteria received neoadjuvant treatment, which could include any form or amount of chemotherapy (with or without radiotherapy) or radiotherapy alone. For each patient, a single representative H & E slide of the tumor bed was selected by the participating pathologists. Slides were selected based on the presence of tumor bed tissue that is typically employed in the College of American Pathologists (CAP) and MD Anderson Cancer Center (MDACC) TRS systems.^9,10^ Participating centers were asked to scan the slides using their own onsite scanners if possible. If onsite scanning was not possible or not preferred, the slides were digitized at Amsterdam UMC using a Philips Intellisite Ultra-Fast Scanner (Philips). All slides were collected anonymously; no clinical or patient-related data were recorded.

### Segmentation and Labeling

For all included cases, specialized gastrointestinal pathologists (J.V. and A.F.) annotated representative, rectangular regions of interest (ROIs) on the whole slide images (WSI) using the ASAP annotation tool.^11^ Different regions per slide were selected to ensure that all kinds of morphology were represented, including both peripheral and central regions of the tumor. ROIs were placed in an area containing RPC, and ideally contained anatomic structures or histologic features considered informative for training a segmentation model. If no RPC could be identified, the ROI was to be placed on areas containing non-neoplastic structures, ideally of ductal origin. A recently developed segmentation model was used to preannotate all the histologic structures of interest as a single class in the ROIs.^8^ The outlines of these annotations were manually checked and, if necessary, corrected by B.V.J. and B.O. Following correction, the WSI and respective annotation files (saved as XML) were distributed among the involved pathologists. Next, the pathologists checked, corrected (if necessary), and classified the annotated structures at the pixel level using Slidescape, an in-house developed annotation software tool.^12^ The segmented structures could be assigned to the following classes: (1) normal ducts; (2) cancer; (3) in situ neoplasia; (4) islets of Langerhans; (5) acinar tissue; (6) atrophic parenchyma/metaplastic ducts; (7) adipose tissue; (8) vessels; (9) nerves; (10) uncertain; (11) lumina; and (12) lymphoid/inflammatory infiltrates. When pathologists were unsure regarding the classification of certain annotations in an ROI (eg, distinguishing normal and pathologic ducts), another participating pathologist was consulted until consensus was reached.

### Data Processing

The ROIs were converted to 24-bit RGB PNG image format at a resolution of 2 px/µm (“20×”). For the same area, ground-truth annotation-based segmentation masks were generated as 8-bit PNG. The original classifications were merged into 4 new classes: (1) non-neoplastic/normal ducts; (2) cancer and in situ neoplasia merged; (3) remaining epithelial structures consisting of islets of Langerhans, acinar tissue, and atrophic/metaplastic parenchyma; and (4) fat. All the remaining classes, that is, vessels, nerves, and lymphoid infiltrates, were treated as background for the current study. Notably, stroma was also categorized as background, being the primary component left after the segmentation of the specified tissues. Cancerous tissue located within boundaries of other structures, for example, vessels or nerves, were separately annotated and regarded as cancerous. Using a sliding window approach, partly (50%) overlapping patches of 512 by 512 pixels were generated from the H & E and corresponding mask images. Patches and associated mask images were only included in the final data set if one of the segmentation classes comprised at least 10% of the patch area. To prevent class imbalance in the training data, the maximum number of patches for each class was set at 50 per case.

### Machine Learning

Semantic segmentation models were developed using U-Net-based architectures.^13^ Specifically, a U-Net model with a DenseNet 161-encoder architecture was utilized. The training process involved the use of binary cross-entropy loss as the loss function and the ADAM optimizer. Data augmentation techniques, including 90-degree, 180-degree, and 270-degree rotations, horizontal and vertical flips, and color jitter,^14^ were applied during the training process. In addition, color normalization was performed to standardize the color distribution of the input data.^15^ To assess the impact of color augmentation and normalization, a comparison was made by training the model without these operations. The model was trained for a total of 30 epochs. If there was no improvement observed in the model’s performance for 7 consecutive epochs, the training process was halted. The machine learning and data handling procedures were implemented using Python 3.7 and PyTorch 1.11.

### Validation Strategy

We implemented a sliding window technique to generate predictions for each patch in the test set. Each patch had a size of 512×512 pixels, with a stride of 256 pixels. Afterward, we combined adjacent patches to form a prediction for the entire ROI. To reduce stitching artifacts, we utilized partially overlapping patches. The final segmentation class of each pixel was determined using a weighted average and then converted into RGB prediction mask images following the methods outlined in the work of Cui et al.^16^ The prediction accuracy of the test set was calculated for each class separately, and the results were expressed as F1 scores.

The model was validated using a by-scanner, internal-external cross-validation.^17^ In this internal-external cross-validation procedure, iteratively, 1 scanner type from the complete data set was left out for validation, and the remainder of the data were used for model development. This process was repeated 4 times until the model was validated for each scanner type (models 1 to 4). After internal-external cross-validation, a final model was constructed based on all data. The performance of each by-scanner internal-external cross-validation was reported as F1 score means and 95% CI. Subsequently, the mean F1 score from each cross-validation was pooled into a single summary value using a random-effects meta-analysis to represent the final model performance across all scanner types. The meta-analytic model consisted of a restricted maximum likelihood estimator with Jackson’s modification of the Hartung-Knapp-Sidik-Jonkman variance correction.^18^ Model performance heterogeneity across scanner types was assessed using the *I* ^2^ and tau^2^ measures and through visual inspection of the forest plots. Meta-analyses were performed in R version 4.2.1 (R Foundation for Statistical Computing), using the “metafor” package.^19^

## RESULTS

### Data Set

A total of 528 patients from 14 centers in 7 countries were included in this study. Of these, 295 (56%) were scanned as iSyntax using a Philips Intellisite Ultra-Fast Scanner and converted to .BigTiff, 146 (27%) were scanned as .NDPI using a Hamamatsu Nanozoomer 2S210, 50 (10%) were scanned as .MRXS with a 3DHistech scanner, and 37 (7%) were scanned as .SVS using Leica scanners. Two slides (Philips, n=1; Hamamatsu, n=1) were excluded due to poor scan quality. Table 1 presents the complete data set, the type of administered neoadjuvant therapy, and the distribution of patient cases across the scanner types. Overall, 119 patients were treated with chemoradiotherapy, and 409 with chemotherapy. Using these patient cases, a total of 1212 ROI were generated, in which 114,960 structures were annotated. The median segmented area for each WSI stood at 3.07 mm² (interquartile range: 2.37 to 7.63). Exemplary segmentations are provided in Supplementary Figure S1, Supplemental Digital Content 1, http://links.lww.com/PAS/B872.

**TABLE 1:** Study Cohort Consisting of 528 Patients with Resected Pancreatic Cancer Following Neoadjuvant Treatment

Center	Country	Scanner	Data format	No. patients	Type of neoadjuvant therapy (n)
Royal North Shore Hospital	Australia	Hamamatsu	.ndpi	146	Chemotherapy: 146 Radiochemotherapy: 0
Amsterdam UMC	The Netherlands	Philips	BigTiff	116	Chemotherapy: 73 Radiochemotherapy: 43
Oslo University Hospital	Norway	3DHistech	.mrxs	50	Chemotherapy: 50 Radiochemotherapy: 0
Leiden University Medical Center	The Netherlands	Philips	BigTiff	38	Chemotherapy: 14 Radiochemotherapy: 24
Erasmus Medical Center	The Netherlands	Philips	BigTiff	34	Chemotherapy: 18 Radiochemotherapy: 16
University Hospital of Duesseldorf	Germany	Leica	.svs	26	Chemotherapy: 24 Radiochemotherapy: 2
Maasstad Hospital	The Netherlands	Philips	BigTiff	23	Chemotherapy: 16 Radiochemotherapy: 7
Isala Hospital	The Netherlands	Philips	BigTiff	22	Chemotherapy: 10 Radiochemotherapy: 12
Asan Medical Center	Korea	Philips	BigTiff	21	Chemotherapy: 21 Radiochemotherapy: 0
Medisch Spectrum Twente	The Netherlands	Philips	BigTiff	14	Chemotherapy: 8 Radiochemotherapy: 6
Koc University	Turkey	Philips	BigTiff	11	Chemotherapy: 11 Radiochemotherapy: 0
MD Anderson Cancer Center	USA	Philips	BigTiff	10	Chemotherapy: 2 Radiochemotherapy: 8
Memorial Sloan Kettering Cancer Center	USA	Leica	.svs	10	Chemotherapy: 10 Radiochemotherapy: 0
OLVG	The Netherlands	Philips	BigTiff	7	Chemotherapy: 6 Radiochemotherapy: 1
Total: 14	7	4	4	528	Chemotherapy: 409 Radiochemotherapy: 119

Four by-scanner internal-external cross-validation models were developed. Model 1 was based on data from 12 centers (n=494) and tested on data from Koc University (Istanbul, Turkey) and Asan Medical Center (Seoul, Republic of Korea) (n=31) to evaluate performance on Philips scanned cases. Model 2 was performed to evaluate performance on Leica scanned cases. This model was trained on data from 12 centers (n=488) and tested on data from Memorial Sloan Kettering Cancer Center (New York City, NY) and the University Hospital of Duesseldorf (Duesseldorf, Germany) (n=37). Model 3 was developed to evaluate performance on 3DHistech-scanned cases. It was trained using data from 13 centers (n=475) and tested on data from the Oslo University Hospital (Oslo, Norway) (n=50). Finally, model 4 was created to evaluate performance on Hamamatsu-scanned cases. It was trained using data from 13 centers (n=382) and tested on data from the Royal North Shore Hospital (Sydney, Australia) (n=146).

### Segmentation Performance

**FIGURE 1:**
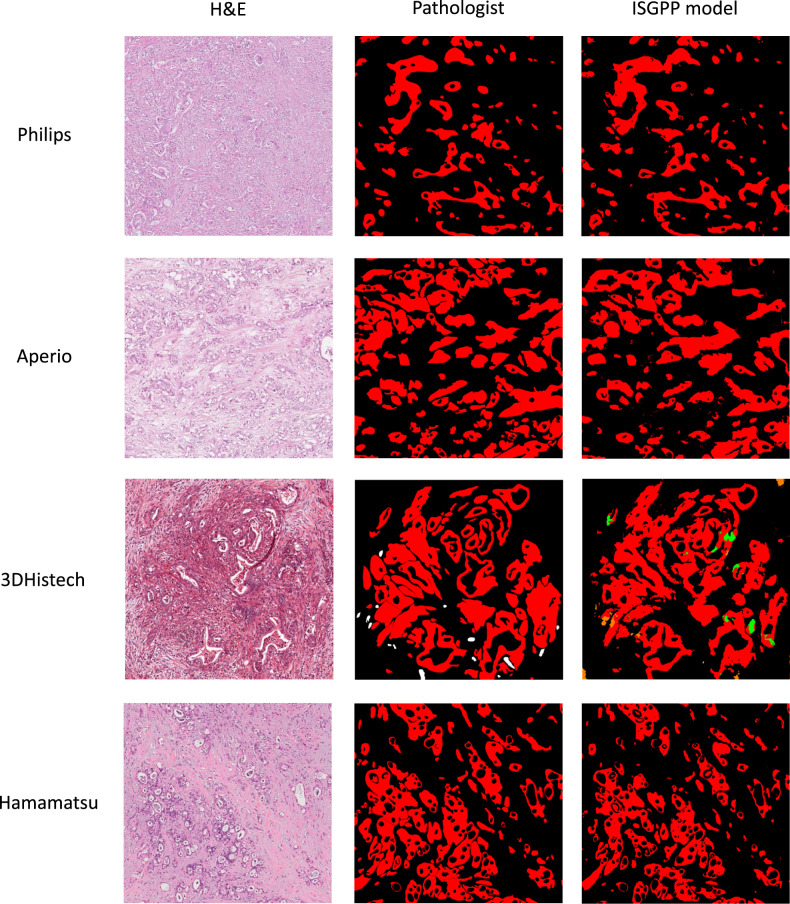
Examples of ISGPP model predictions versus pathologist-based ground-truth annotations. Cases with median F1 scores for each by-scanner cross-validation. The Philips example is a case from South Korea with an F1 score of 0.83. The Leica example is a case from Germany with an F1 score of 0.79, and the 3DHistech example is a case from Norway with an F1 score of 0.80. Lastly, the Hamamatsu example is a case from Australia with an F1 score of 0.83. Red represents cancerous ducts and in situ neoplasia, black represents the background, green represents noncancerous ducts, white represents uncertain structures, and orange represents remaining noncancerous tissue.

**FIGURE 2:**
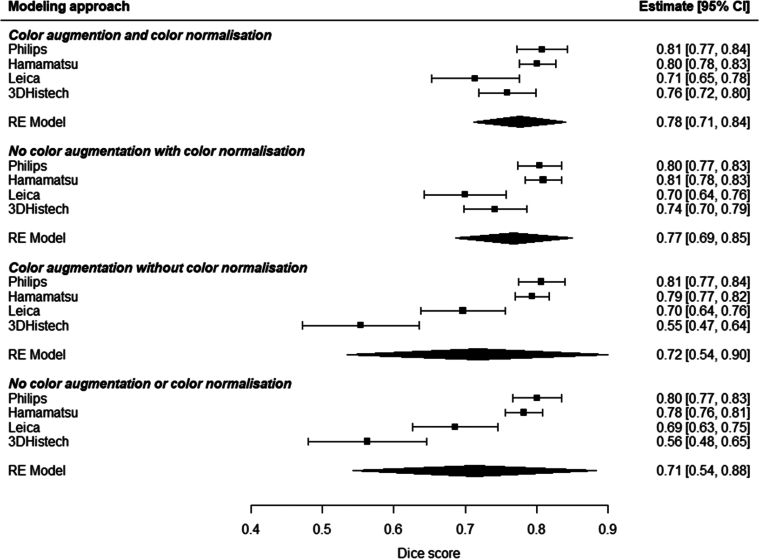
Forest plots of model performance represented as F1 scores. Forest plots showing the effect sizes and corresponding 95% CIs for the 4 subgroups of studies investigating the impact of different scanners, normalization, and augmentation on the F1 score. The color normalized and color augmented subgroup is shown in the top section, followed by the normalized, augmented, and subgroups wherein these techniques are not applied, respectively. The diamond at the bottom of each subgroup represents the overall effect size estimate, and its width corresponds to the 95% CI. The mean effect size for each subgroup is also provided.

The pooled F1 score across all scanner types was 0.78 (95% CI, 0.71-0.84, *I* ^2^=73.8% [0.0-98.6], Tau^2^=0.001 [0.0-0.025]) in the meta-analysis. The performance of the segmentation model varied across scanner types (Fig. 2 and Supplementary Table 1, Supplemental Digital Content 2, http://links.lww.com/PAS/B873). The highest F1 score was observed when validating the model in the set of Philips scanned cases (n=31; mean F1 score, 0.81 [95% CI, 0.77-0.84]). Model performance was lower at external validation in the Hamamatsu-scanned cases (n=146; mean F1 score, 0.80 [0.78-0.83]) and the 3DHistech-scanned cases (n=50; mean F1 score, 0.76 [0.72-0.80]). The lowest F1 score was observed in the set of Leica scanned cases (n=37), with a mean F1 score of 0.71 (0.65-0.78). Figure 1 provides visual examples of the model performance. Figure 2 shows forest plots illustrating the cross-validations and the meta-analysis. Supplementary Table S1, Supplemental Digital Content 2, http://links.lww.com/PAS/B873 details all analyses.

### Subanalyses—Color Augmentation and Normalization

The application of both color augmentation and normalization yielded the highest mean F1 scores, with a pooled F1 score of 0.78 (0.71-0.84, *I* ^2^=73.8% [0.0-98.6], Tau^2^=0.001 [0.0-0.025]), and scanner-specific scores of 0.81 (0.77-0.84) for Philips, 0.71 (0.65-0.78) for Leica, 0.76 (0.72-0.80) for 3DHistech, and 0.80 (0.78-0.83) for Hamamatsu. When only applying normalization, relatively similar results were obtained, with a pooled F1 score of 0.77 (0.69-0.84, *I* ^2^=86.3% [53.4-99.1], Tau^2^=0.002 [<0.001-0.04]). Here, scanner-specific scores were 0.80 (0.77-0.84) for Philips, 0.70 (0.64-0.76) for Leica, 0.74 (0.70-0.79) for 3DHistech, and 0.81 (0.78-0.83) for Hamamatsu. However, with only augmentation, or with neither technique, model performance was relatively lower. When only using augmentation, the pooled F1 score was 0.72 (0.54-0.90, *I* ^2^=96.6% [88.3-99.8], Tau^2^=0.01 [0.003-0.19]). The scanner-specific scores were 0.81 (0.77-0.84) for Philips, 0.70 (0.64-0.76) for Leica, 0.55 (0.47-0.64) for 3DHistech, and 0.79 (0.77-0.82) for Hamamatsu. Similarly, without these techniques, the pooled F1 score was 0.71 (0.54-0.88, *I* ^2^=95.7% [85.4-99.7], Tau^2^=0.01 [0.003-0.16]), and individual scanner scores were 0.80 (0.77-0.84) for Philips, 0.69 (0.63-0.75) for Leica, 0.56 (0.48-0.65) for 3DHistech, and 0.78 (0.76-0.81) for Hamamatsu. Overall, color augmentation and normalization, specifically the latter, appeared to mainly affect 3DHistech-scanned cases: with normalization: mean F1 score of 0.77 (0.69-0.85); without normalization: mean F1 score of 0.55 (0.47-0.64). Figure 2 shows forest plots illustrating the cross-validations and the meta-analysis. Supplementary Table S1, Supplemental Digital Content 2, http://links.lww.com/PAS/B873 details all analyses.

### Model Availability

The final model is published on https://github.com/PHAIR-Consortium.

## DISCUSSION

This study describes the first international, multicenter, by-scanner internally-externally cross-validated segmentation model for RPC after NAT. With a pooled F1 score in the meta-analysis of 0.78 (95% CI, 0.71-0.84), the presented ISGPP model can accurately segment and quantify RPC. By doing so, it lays the essential groundwork for clinically relevant quantitative assessments of NAT response evaluation in PDAC. The model is published alongside this study for public use.

Upon analyzing all the developed models, several important aspects related to modeling techniques were observed. The individual cross-validations revealed lower mean F1 scores for the Leica and 3DHistech test sets (0.71 [0.65-0.78], 0.76 [0.72-0.80]) compared with the Philips and Hamamatsu test sets (0.81 [0.77-0.84], 0.80 [0.78-0.83]). In addition, a notable finding was the strong benefit of the color normalization technique for the 3DHistech-scanned cases, resulting in an improved F1 score of 0.77 (0.69-0.85) compared with cases without normalization, which achieved an F1 score of 0.55 (0.47-0.64). These results indicate that the final model may encounter more challenges in accurately segmenting cases scanned using Leica and 3DHistech scanners. These performance discrepancies could be attributed to variations in the scanners themselves, differences in staining protocols employed by various laboratories, and enough comparable cases within the training data. These factors are known to be influential in the field of digital pathology.^20^ Visual comparison of the slides also revealed noticeable differences in color profiles between cases scanned on Leica and 3DHistech versus Philips and Hamamatsu scanners (see Supplementary Figure S2, Supplemental Digital Content 3, http://links.lww.com/PAS/B874). It is, however, important to note that these color differences are not only the result of the scanner brand and settings but also stem from variations in staining protocols. When applying the final model, users should be aware of these performance differences, and researchers should recognize the value of techniques such as normalization and augmentation in enhancing model generalizability.

The present model compares favorably to the first version in several aspects.^8^ First, the study sample consists of a considerably larger number of unique patient cases (n=528 vs. n=65) and does not exclude cases deemed too difficult to segment. Second, the study includes more diverse data, with cases from 14 different laboratories in 7 countries, rather than only cases from the Amsterdam UMC. Finally, the study includes data collected using 4 different scanner types, providing a more comprehensive evaluation of model performance across a range of settings. These improvements enhance generalizability and provide a more robust assessment of its performance in diverse clinical contexts.

**FIGURE 3:**
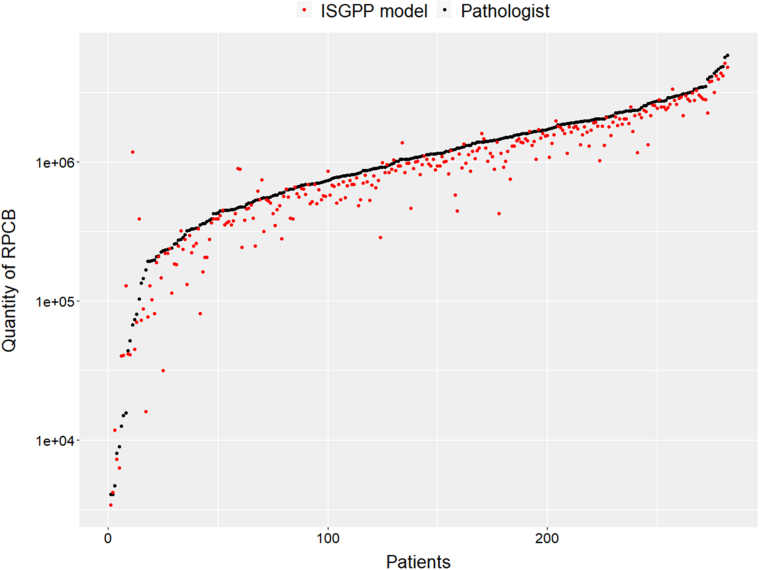
Comparing AI RPC quantification to reference standard in the cross-validations. Logarithmic graphs are used to compare the number of pixels designated as RPC by the AI model (red) and the pathologist (black) for patients in the combined cross-validation sets. The *y-*axis indicates the number of pixels designated as RPC, while the *x*-axis shows the cases in order of ascending RPC according to the pathologist’s assessment. This visualization allows for a comparison of the performance of the AI model and the pathologist in quantifying RPC in the test set.

Several limitations should be taken into account when interpreting the results of the present study. First, the study sample, while larger and more diverse than in previous work, may not be representative of the overall population. The data set is disproportionately composed of Dutch and Australian patients and scanners, comprising ~75% of the cohort. This may have contributed to the relatively better mean F1 scores with narrower 95% CIs and limited benefit from color augmentation and normalization techniques. It is uncertain how the models would perform on populations not included in the study sample. Moreover, the validation cohorts included only a few cases treated with radiotherapy, further complicating our understanding of the model’s performance in such cases. However, it is worth noting that for these 2 patients, the mean Dice score was 0.79. Second, bias may exist in the selection of ROIs. The ROIs selected for analysis were based on the presence of useful training data rather than representing the entire slide. The content and size of the ROIs also varied, due to the heterogeneous presentation of treated PDAC. Differences in PDAC morphology after NAT were included in the ROIs as well as in peripheral and central areas of the tumor. However, it is possible that the reported results might not accurately reflect model performance on whole slides, which could contain tissue types or less frequent morphologies not included in the ROIs. Furthermore, the presence of in situ neoplasia differed between slides. How to deal with high-grade in situ neoplasia when quantifying residual tumor is challenging as it is almost impossible to differ morphologically high-grade PanIN and cancerization of ducts. Still, it is important to note that manually annotating complete WSIs at the pixel level is practically impossible. To tackle this issue, future studies should consider a random selection of (multiple) ROIs. Third, the quality of the reference standard used in the study may have been impacted by interobserver variation and bias. These issues may have led to suboptimal segmentations and labels. Upon inspection of outliers in terms of model performance in the test set, these issues were present, particularly in cases where the classification of structures as non-neoplastic ducts, in situ neoplasia, or cancer was uncertain or when neoplastic ducts were located amidst heavily inflamed areas, obscuring boundaries between structures. We anticipate that these issues will have a limited influence on future work aimed at developing an RPC quantity TRS system, but emphasize that future studies should aim to improve the subclassification of these categories. In addition, the segmentation models consistently provided a lower estimate of RPC compared with the human assessor (Fig. 3). This discrepancy potentially originates from the human assessor having a systematic bias in the form of providing less accurate segmentations regarding the boundaries of the malignant duct structures. These issues likely affected the reliability of the achieved F1 scores. Overall, this study’s primary strength lies in its extensive data set, featuring patients from various countries, WSI from various laboratories, and scanner types, thus providing a robust model for the segmentation of RPC after NAT.

Future research should focus on improving segmentation generalizability in various settings. Given the relatively suboptimal performance for the Leica and 3DHistech-scanned cases, and the presence of outliers in terms of poor model performance for all test sets, additional improvement may be realized. This study utilized a U-net architecture with a DenseNet 161 encoder, selected through a series of pilot experiments comparing different encoders, including ResNet152, EfficientNet-B4 and -B7, and InceptionV4. The results indicated that DenseNet 161 achieved the highest F1 scores (data not shown). However, it is worth exploring alternative architectures such as transformers,^21^ which have recently become available and could also be evaluated in future studies. Another sensible step would be the incorporation of more high-quality data into model development. This should involve the inclusion of data from different laboratories using different scanners. Optionally, techniques such as antibody-supervised learning could be explored to increase reference standard quality.^22^

In terms of value for response evaluation, the next step is to investigate the model's conversion into a clinically relevant TRS system by providing new definitions for response. This would involve investigating the correlation between RPC quantities and survival, and an in-depth comparison with commonly established TRS systems such as the CAP and MDACC systems. Although the segmentation model allows for quantification of RPC in a slide, the extent of sampling that is required for response evaluation, taking into account the heterogeneity of tumor response, remains unclear.^5,23,24^ Moreover, despite extensive sampling, it is unclear how RPC needs to be calculated for a given patient. Multiple approaches could be used, among which are, for example, calculating the total sum of all RPC pixels over all slides or the average RPC over all slides. Second, when an improved system is established, it should be validated retrospectively and prospectively in a diversity of clinical settings. Furthermore, it is worth noting that while an AI-based TRS system that quantifies RPC in terms of a number of pixels is a significant advancement in the objective evaluation of therapeutic response, it may be limited by its focus on RPC quantification alone. Other factors, such as the enumeration of cancer cells or geometric characterization of cancer cells, may provide additional valuable information for response assessment.^25^ Similarly, the quality and composition of surrounding tissue may offer critical insights.^26,27^ Future AI-based methods should, therefore, delve into the significance of data from the entire tumor bed for a comprehensive evaluation of therapeutic responses.^28,29^ This includes examining stromal features and lymphoid infiltrates, which are essential for understanding the full context of the tumor environment.

In summary, the model presented in this study demonstrates strong performance in segmenting RPC, with a by-scanner internally-externally cross-validation F1 score of 0.78 (0.71-0.84). By enabling automated RPC segmentation, the ISGPP model forms the basis for quantification of the RPC as a more objective approach to evaluating the response to NAT. The ISGPP model is available for public use.

## Supplementary Material
