# Supplementary material for: Artificial Intelligence-based Segmentation of Residual Pancreatic Cancer in Resection Specimens Following Neoadjuvant Treatment (ISGPP-2): International Improvement and Validation Study
Source: Am J Surg Pathol. 2024 Jul 2;48(9):1108–16. doi: 10.1097/PAS.0000000000002270 (PMC11321604; doi:10.1097/PAS.0000000000002270)
Supplement: Supplementary file 1 [file pas-48-1108-s001.docx]

**Supplementary Figure S1:** Sample annotations for clarity on annotation precision

**
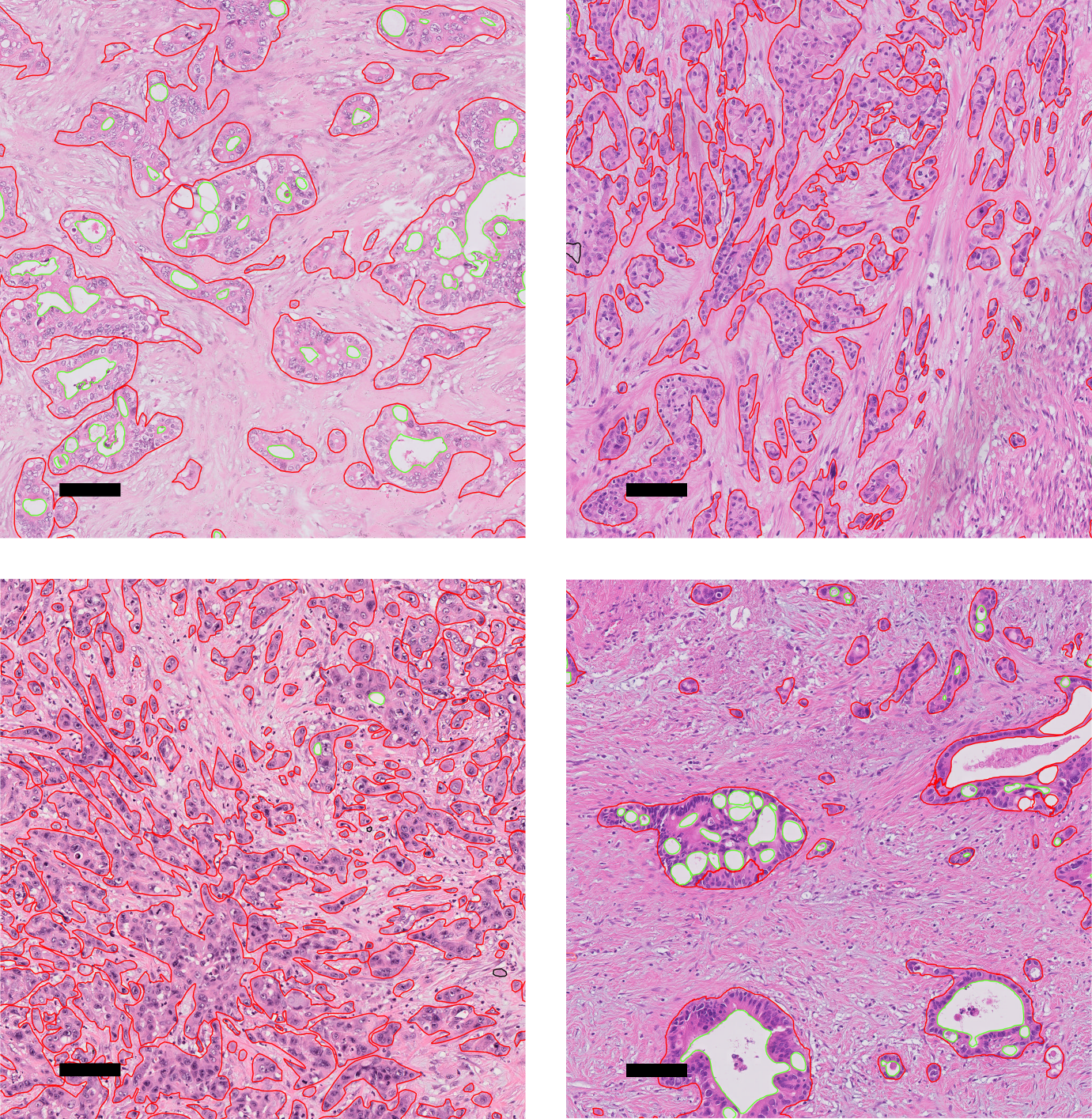
**

**Legend:** This figure presents four illustrative examples of sample annotations, aimed at elucidating the methodology and precision of the annotation process. A black scale bar, representing a length of 100 μm, is provided for reference. Cancerous epithelium annotations are depicted in red, while green annotations denote luminal structures.
