# Supplementary material for: Artificial Intelligence-based Segmentation of Residual Pancreatic Cancer in Resection Specimens Following Neoadjuvant Treatment (ISGPP-2): International Improvement and Validation Study
Source: Am J Surg Pathol. 2024 Jul 2;48(9):1108–16. doi: 10.1097/PAS.0000000000002270 (PMC11321604; doi:10.1097/PAS.0000000000002270)
Supplement: Supplementary file 2 [file pas-48-1108-s002.docx]

**Supplementary Table S1:** Segmentation performance of various modeling approaches

| Modeling appproach | Mean F1 | 95% CI | I^2^ ( 95% CI) | Tau^2^ (95% CI) | | |
| --- | --- | --- | --- | --- | --- | --- |
| Color augmention and color normalisation | | |  |  | | |
| Meta-analysis | 0.78 | 0.71 – 0.84 | 73.8% (0 – 98.6) | 0.001 (0.0 – 0.03) | | |
| Philips | 0.81 | 0.77 – 0.84 |  |  | | |
| Leica | 0.72 | 0.66 – 0.77 |  |  | | |
| 3DHistech | 0.76 | 0.72 – 0.80 |  |  | | |
| Hamamatsu | 0.80 | 0.78 – 0.83 |  |  | | |
|  |  |  |  |  | | |
| No color augmentation with color normalisation | | |  |  | | |
| Meta-analysis | 0.77 | 0.69 – 0.85 | 86.3% (53.4 – 99.1) | 0.002 (<0.001 – 0.04) | | |
| Philips | 0.80 | 0.77 – 0.84 |  |  | | |
| Leica | 0.70 | 0.64 – 0.76 |  |  | | |
| 3DHistech | 0.74 | 0.70 – 0.79 |  |  | | |
| Hamamatsu | 0.81 | 0.78 – 0.83 |  |  | | |
|  |  |  |  |  | | |
| Color augmentation without color normalisation | | |  |  | | |
| Meta-analysis | 0.72 | 0.54 – 0.90 | 96.6% (88.3 – 99.8) | 0.01 (0.003 – 0.19) | | |
| Philips | 0.81 | 0.77 – 0.84 |  |  | | |
| Leica | 0.70 | 0.64 – 0.76 |  |  | | |
| 3DHistech | 0.55 | 0.47 – 0.64 |  |  | | |
| Hamamatsu | 0.79 | 0.77 – 0.82 |  |  | | |
|  |  |  |  |  | | |
| No color augmentation or color normalisation | | | | | | |
| Meta-analysis | 0.71 | 0.54 – 0.88 | 95.7% (85.4 – 99.7) | 0.01 (0.003 – 0.16) | | |
| Philips | 0.80 | 0.77 – 0.84 |  |  | | |
| Leica | 0.69 | 0.63 – 0.75 |  |  | | |
| 3DHistech | 0.56 | 0.48 – 0.65 |  |  | | |
| Hamamatsu | 0.78 | 0.76 – 0.81 |  |  | | |
| **Legend:** F1 score (range: 0.0 – 1.0), 95% CI = 95% confidence interval | | | | |  |  |
