# Supplementary material for: Artificial Intelligence-based Segmentation of Residual Pancreatic Cancer in Resection Specimens Following Neoadjuvant Treatment (ISGPP-2): International Improvement and Validation Study
Source: Am J Surg Pathol. 2024 Jul 2;48(9):1108–16. doi: 10.1097/PAS.0000000000002270 (PMC11321604; doi:10.1097/PAS.0000000000002270)
Supplement: Supplementary file 3 [file pas-48-1108-s003.docx]

**Supplementary Figure S2:** Color profile comparison of by-scanner subgroups


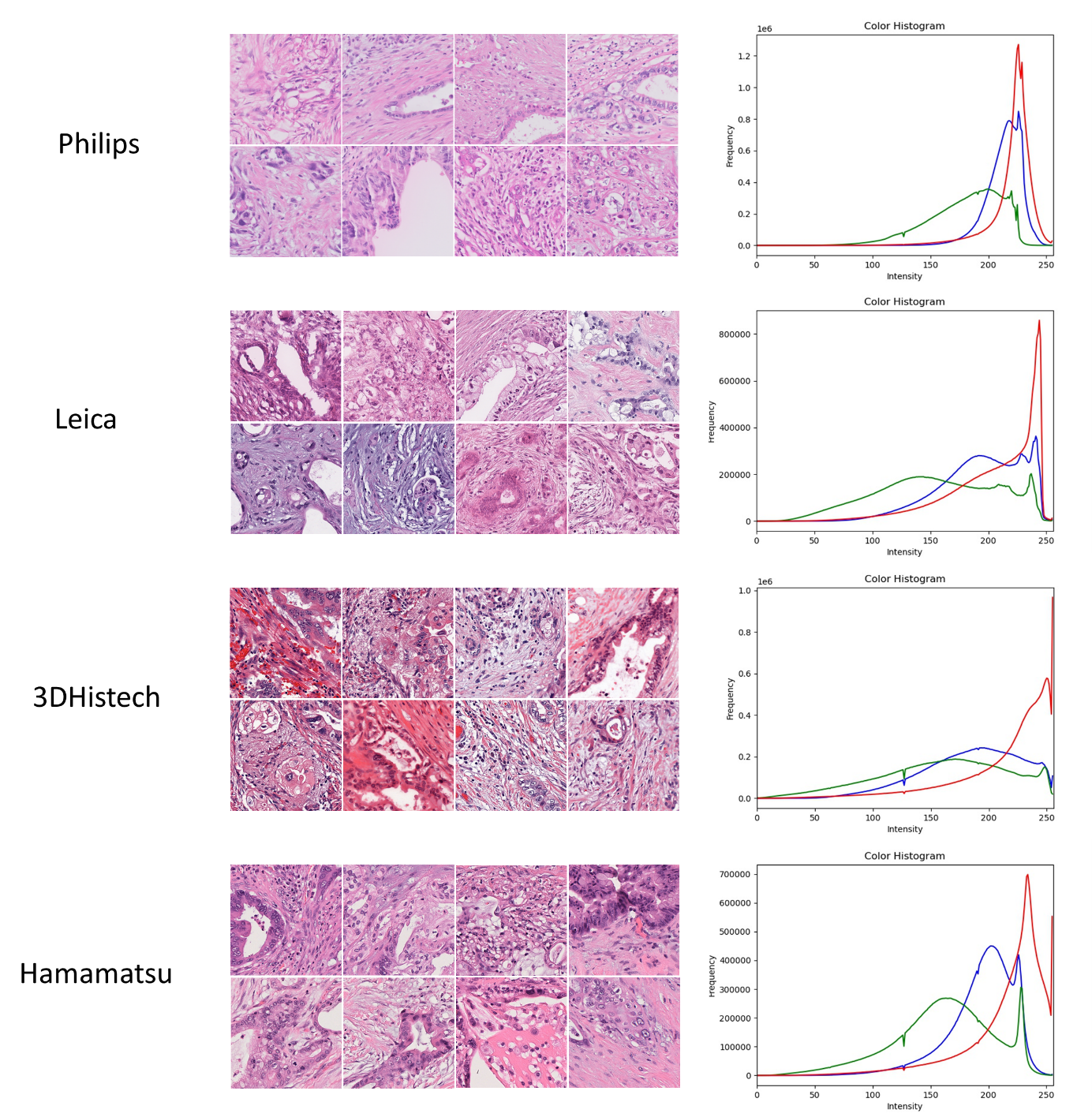


**Legend:** This figure displays a comparison of color profiles among subgroups, categorized by scanner type. Within each subgroup, eight random patches containing tumor tissue are presented, allowing a visual assessment of the color variations. Additionally, a color histogram is provided, illustrating the color profile derived of 100 randomly selected and aggregated patches. The X-axis represents the intensity values of each RGB channel found in pixels, ranging from 0 minimum) to 255 (maximum) intensity. The Y-axis indicates the frequency of occurrence for pixels with specific intensity values. The red, blue, and green lines correspond to the intensity values for the respective red, blue, and green channels of the RGB color model.
